# Supplementary material for: Dynamic mitochondrial transcription and translation in B cells control germinal center entry and lymphomagenesis
Source: Nat Immunol. 2023 Apr 24;24(6):991–1006. doi: 10.1038/s41590-023-01484-3 (PMC10232359; doi:10.1038/s41590-023-01484-3)
Supplement: Supplementary file 1 — Supplementary Methods. [file 41590_2023_1484_MOESM1_ESM.pdf]

nature immunology

Article https://doi.org/10.1038/s41590-023-01484-3

Dynamic mitochondrial transcription and translation in B cells control germinal center entry and lymphomagenesis

In the format provided by the authors and unedited

Supplementary methods

NP conjugation

NP-APC conjugation was performed as described[1], with certain modifications. Briefly, one mg of natural allophycocyanin protein (APC) (Stratech Scientific Ltd) was transferred into Slide-A-Lyzer™ MINI Dialysis Device, 3.5K MWCO (Thermo Fisher Scientific, cat: 69550) and dialyzed for 5 hours, overnight, then for 4 hours in 1L 3% $NaHCO_3$ at 4°C. NP-Osu (Biosearch, cat no: N-1010-100) was dissolved in dimethylformamide (Merck) to a concentration of 10mg/ml while vortexing. The NP-Osu was added to the dialyzed APC at a ratio of 20µg:1 mg (NP-low conjugation) and 80µg:1 mg (NP-high conjugation) and rotated at 20°C for 2 hours, protected from light. The NP-APC conjugates were then dialyzed in 1L 3% $NaHCO_3$ at 4°C overnight, then 1L PBS overnight. NP probes were stored at 4°C in the dark until use. NP-APC conjugates were used at 1:100 dilution. For memory B cell detection, NP_{Hi}-APC was used. Directly conjugated NP-PE was purchased from Biosearch and used at 1:200.

ELISA

96 well EIA/RIA plates (Corning, cat: 3590) were coated with NP-BSA (NP_{1-4} or $NP_{<20}$, cat: N-5050XL-10 and cat: N-5050H-10) at 5µg/ml in bicarbonate/carbonate coating buffer overnight at 4°C. The next day, plates were washed with PBS and blocked with 5% skimmed milk in PBS for 2.5 hours at 37°C. Sera obtained from mice (NP-CGG-immunized day 14 & 49) were serially diluted in 1% skimmed milk and incubated in blocked plates at serial dilutions for 1 hour at 37°C. After multiple washes with PBS supplemented with 0.05% Tween-20, alkaline phosphatase-conjugated goat anti-mouse IgG1(Southern Biotech cat: 1071-04) or IgM (Southern Biotech, cat: 1021-04) detection antibodies were added (1:2000) and incubated for 1 hour at 37°C. After the final washing step, plates were developed with alkaline phosphatase substrate (Sigma, cat: P7998-100ML) for 10-30 mins and read on a FLUOstar Omega plate reader at 5 min intervals.

STED microscopy

STED super resolution imaging was performed using a Leica TCS SP8 laser scanning STED system equipped with an oil objective (HC PL APO 100× NA 1.40) and a 775nm depletion laser. Isolated naïve and GC B cells labelled with anti-TFAM (Abcam) and Alexa Fluor 647 conjugated goat anti-rabbit secondary antibody (Thermo Fisher Scientific, cat: A55055) were imaged in confocal and STED mode sequentially. COXI labelled with AF488 was subsequently imaged in confocal mode to define mitochondrial boundaries. Acquired STED images were deconvoluted using Deconvolution Express mode with Standard setting in Huygens Essential software (v22.04) (Scientific Volume Imaging, Hilversum, Netherlands) and exported using Image J software.

Image analysis

Zen Blue (v3.4, ZEISS) and ImageJ software were used for image analysis/processing. For the determination of GC properties in splenic sections, tdTomato and/or GL-7 signals were used as a reference to identify GCs, and IgD for naïve B cell follicles. Defined areas were introduced as regions of interest (ROIs) using the Analyze Particles function. Mitochondria were segmented based on Mito-QC GFP or COX I signal, and subsequently area and signal intensity calculations were performed using the Analyze Particles function in ImageJ. For 3D

volumetric analyses of mitochondria, the 3D Objects Counter function was used. Mitochondrial 5-EU incorporation is quantified first by identifying mitochondrial area based on thresholded COXI signal. Background noise in the 5-EU channel was removed using the Subtract Background (Rolling ball radius 20 pixels) function. Areas outside of the mitochondrial ROIs were removed, and the remaining 5-EU integrated density was determined by the 3D Objects Counter function. 3D TFAM-mitochondrial nucleoid complexes were enumerated by 3D Suite[2]. Briefly, local maxima of TFAM signals were determined for each z-slice using 3D Fast Filters (kernel x, y and z 1px each) which were then inputted into a 3D Spot segmentation module with local thresholded Gaussian fit (Radius max 10 px, SD value 1.50).

PC-PB quantification on splenic IHC images was performed in ImageJ software. Confocal images were imported in split channel mode. For total splenic section area quantification, the DAPI channel was thresholded (using Yen setting) and measured via the Analyze Particles function (20µm size threshold + include holes). Then, a Gaussian blur filter (2 sigma radius) was applied to CD138, tdTomato and Blimp1-mVenus channels followed by Background subtraction (rolling ball radius 50px). Subsequently, each channel was autothresholded (Yen) and Watershed segmentation was applied to thresholded binary images. CD138 and tdTomato channels were inputted into Image calculator using the AND function. The resulting image was then inputted into the same mode, including Blimp1-mVenus channel. The final image was subsequently quantified using the Analyze Particle mode (0.5µm size threshold + Include holes) to identify the triple positive PC fraction. PB numbers were then calculated as follows: $CD138^+$ Blimp1-$mVenus^+$ cell number minus triple positive ($CD138^+$ Blimp1-$mVenus^+$ $tdTomato^+$) PC number. For T_{FH} enumeration on splenic sections, first $tdTomato^+$ GC clusters were auto-thresholded (Otsu). GC area was calculated using the Analyze Particle function. After brightness/contrast adjustments, $CD3^+$ T_{FH} cells within GC ROIs were manually counted.

For DZ/LZ analyses in tissue sections, the nesting function in Zen Blue was used by identifying $tdTomato^+$ GCs as ROIs. The CD21/35 signal was then used to calculate the area of the LZ. Normalized DZ area was quantified as follows: (GC area – LZ area) / GC area. Quantification was performed for each individual GC pooled from splenic sections. GCs with values >0.95 were excluded as not including any representative LZ area. Airyscan reconstruction was performed in Zen Blue software with Medium filter setting for images acquired with the relevant module. ImageJ Macro codes used for image analyses are available upon request.
When GC B cells were isolated from Aicda-Cre × $Rosa26^{STOP}$tdTomato mice, the tdTomato reporter signal was used to filter out contaminating non-GC B cells.

For image analysis of the UPR^{mt}, the integrated density of LONP1 was first calculated using the 3D Objects Counter function and normalized to the TOMM20 integrated density obtained from the same cell.

In vitro mouse primary B cell culture

B cells were isolated from spleens of unimmunized $Tfam^{loxP}$ and wild type mice carrying the Rosa26-stop-tdTomato allele without Aicda-Cre using the Pan B cell isolation kit II (Miltenyi). TFAM was deleted by TAT-Cre recombinase (66.6 U/ml, approx. 1.5µM, Merck, cat: SRC508) in serum-free complete RPMI (supplemented with 1× GlutaMAX, 1mM Sodium pyruvate, 10mM HEPES, 50 U/ml Penicillin/Streptomycin and 50µM 2-Mercaptoethanol, pH = 7-7.4) for 45 mins at 37°C and 5% CO_2. CellTrace Violet dye (CTV, Thermo Fisher

Scientific) at 10μM was added directly at the 30th min of TAT-Cre incubation and cells were incubated for an additional 15 min at 37°C with CTV and TAT-Cre. Subsequently, cells were washed three times with 10% FCS containing complete RPMI, and live cells were counted manually using Trypan Blue exclusion of dead cells. B cells were cultured in U-bottom 96-well plates at a concentration of 1×10^6/ml with anti-IgM (1μg/ml, Jackson Immuno), anti-CD40 (1μg/ml, Miltenyi Biotec) and IL-4 (40ng/ml, Peprotech) stimulation in complete RPMI for four days at 37°C in a humidified incubator with 5% CO_2.

T_{FH}-B cell co-culture

The method was described by Sage et al[3]. Briefly, $CD19^-CD4^+CXCR5^{hi}ICOS^+GITR^-$ T_{FH} cells were isolated from SRBC-immunized (enhanced protocol) WT mice at day 14. Naïve B cells were isolated from *Tfam*^{loxP} and wild type mice, both carrying the Rosa26^{STOP}tdTomato allele with anti-CD43 microbeads (Miltenyi Biotec). Following TAT-Cre treatment (~1.5μM, 45 min), 5×10^4 wildtype or Tfam$^{-/-}$ B cells were co-cultured with 3×10^4 T_{FH} cells in U bottom 96 well-plate in the presence of anti-CD3 (2μg/ml) (145-2C11, Thermo Fisher Scientific, cat: 16-0031-82) and anti-IgM (5μg/ml, Jackson Immuno) for 6 days. At the end of the culture period, the percentages of $CD19^+$ GL-7$^+$ IgG1$^+$ tdTomato$^+$ in vitro GC B cells were quantified via flow cytometry.

Peptide presentation and T-B conjugate assay

The technique was performed as previously described[4,5] with modifications. CD45.1$^+$ GFP$^+$ OT-II CD4$^+$ T cells were purified from mouse spleens using magnetic beads (CD4 T cell MojoSort isolation kit, BioLegend) and activated with 10μg/ml recombinant mouse IL-2 IS (Miltenyi, 130-120-662) for 3 days in 24-well plates coated with 3μg/ml anti-CD3 (145-2c11; BioLegend) and 3μg/ml anti-CD28 (37.51, BioLegend). Naïve B cells were isolated from Rosa26^{STOP}tdTomato × Tfam WT or Tfam^{loxP} mice using anti-CD43 micro-beads (Miltenyi Biotec), and following TAT-Cre administration, activated for 4 days using the iGB-40LB in vitro system in 6 well plates. On day 4, fibroblasts were removed, and 2×10^5 purified iGB cells were pulsed with ovalbumin peptide (amino acids 323–339) (Genescript) at varying concentrations (0μM, 0.1μM and 1μM) for 30 min at 37°C in 96 well U-bottom plates (Falcon) in triplicates per condition. After the addition of 5×10^5 activated OT-II T cells to each well, the plates were centrifuged at 500g for 5 min at 20°C. The centrifuged cell pellets were then incubated for 30 min at 37°C. 16% PFA was added directly to the wells to achieve a final concentration of 4%, and the pellets were gently resuspended. The cells were fixed for 10-15 mins and directly acquired using an Aurora spectral flow cytometer (Cytek) at low flow rate setting to minimize artificial doublets. 15,000 events were recorded per condition. Before acquisition, the plate was shaken at 1100rpm by the plate holder. As a negative control, T and B cells were mixed in the absence of OVA peptide without centrifugation and subsequent incubation.

Cytoplasmic calcium assay

Due to the time and temperature sensitive nature of calcium dyes, experiments were performed in four batches such that one wild type and one B-Tfam mouse spleen were processed in each replicate. Single cell suspensions were prepared from spleens and 2×10^6 cells were placed in V bottom 96 well plates, then labelled with fixable viability dye and then anti-B220 antibody. Subsequently, cells were stained with the cytoplasmic calcium indicator dye Fluo-4 AM (Thermo Fisher Scientific, cat: F14201) at 10μg/ml in Iscove's Modified Dulbecco's Media (IMDM) supplemented with 1% FBS at 37°C for 15 mins. Following a single wash, cells were resuspended in 400μl warm IMDM with 1% FBS. Samples were kept

in a warm water container (~37°C) throughout the flow cytometry acquisition to maintain physiologic activity. Baseline fluorescence intensity was measured for 30s prior to stimulation with CXCL12 (200ng/ml) or anti-IgM (10µg/ml). Flow cytometry acquisition was performed on a BD Fortessa X20 at low-speed setting for 5 min. The acquisition sequence was alternated between wild type and experimental samples in each batch to avoid potential timing-related noise. Analyses were performed using the Kinetics module in Flow Jo software.

Lymphoma adoptive transfer

Following the manifestation of clinical signs of lymphoma, Eµ-Myc mice were sacrificed and spleens were harvested. Non-B cells were depleted using the Pan B cell Isolation Kit II (Miltenyi Biotec) to enrich lymphoma cells. 2×10^6 lymphoma cells were then intravenously injected into recipient B6.SJL.CD45.1 mice in 200µL PBS. Recipient mice were sacrificed at 3 weeks following adoptive transfer, and inguinal lymph nodes were harvested for further analyses.

Mixed bone marrow chimera generation

B6.SJL.CD45.1 recipient mice were given two doses of 5.5Gy irradiation four hours apart. Mice were then intravenously injected with 4×10^6 mixed bone marrow (BM) cells at a 1:1 ratio, isolated from age- and sex-matched $CD45.2^+$ Aicda-WT and $CD45.1^+$ WT or $CD45.2^+$ Aicda-Tfam and $CD45.1^+$ WT donor mice. Recipient mice were maintained on antibiotics (Baytril, Bayer corporation) in drinking water for two weeks. BM reconstitution was confirmed via flow cytometry of peripheral blood at 8 weeks. Mice were immunized with SRBC at 11 weeks and spleens were analyzed at day 7 post immunization.

Quantification of metabolism by protein translation (SCENITH)

This technique was performed as described, with modification[6]. Briefly, splenocytes were split into 4 groups and incubated for 30 mins with or without metabolic inhibitors (1µM oligomycin and/or 100mM 2DG, both from Merck) in 96 well plate at 37°C. The alkynylated puromycin analog OPP (20µM final concentration, Thermo Fisher Scientific) was then directly added to the wells for an additional 30 mins incubation. Subsequently, cells were washed and labelled with Live/Dead viability dye and surface antibodies, after which they were fixed with 4% PFA. Click Chemistry labelling was performed according to Click-iT™ Plus OPP Alexa Fluor 647 Protein Synthesis Assay Kit (Thermo Fisher Scientific, cat: C10458) following permeabilization. The percentages of mitochondrial dependence, glycolytic capacity, glucose dependence and FAO/AAO (fatty acid oxidation and amino acid oxidation) were measured using the gMFI values of OPP-AF647 from cells treated with 2-deoxyglucose (2-DG), oligomycin, or 2-DG and oligomycin. The formulae are summarized below:

Glucose dependence (%): $= 100 \times [(OPP_{\text{No inhib}} - OPP_{\text{2DG}}) / (OPP_{\text{No inhib}} - OPP_{\text{(2DG \& oligomycin)}})]$
FAO/AAO capacity (%) = 100 - (Glucose Dependence)
Mitochondrial dependence (%) $= 100 \times [(OPP_{\text{No inhib}} - OPP_{\text{oligomycin}}) / (OPP_{\text{No inhib}} - OPP_{\text{(2DG \& oligomycin)}})]$
Glycolytic capacity (%) = 100 - (Mitochondrial Dependence)

Extracellular flux analysis

Real-time oxygen consumption rates (OCR) and extracellular acidification rates (ECAR) were recorded using a Seahorse XFe96 Extracellular Flux Analyzer (Agilent). Briefly, ex vivo isolated naïve or overnight stimulated (anti-CD40 at 5µg/ml and IL-4 at 1ng/ml) 3×10^5 B cells were plated on a poly-D lysine (PDL) (Sigma)-coated XF96 cell culture microplate and incubated at 37 °C for a minimum of 30 min in a CO_2-free incubator in assay medium (XF RPMI medium pH 7.40 supplemented with 2 mM L-glutamine, 1 mM pyruvate and 10 mM glucose). iGB cells were rested in the presence of IL-4 at 1ng/ml overnight after detachment from a fibroblast feeder layer on day 4 of the iGB culture. The next day, they were plated at 2×10^5 per well density on PDL-coated XF96 cell culture microplate. Basal OCR and ECAR were measured, then followed by the addition of the MitoStress Test inhibitors: oligomycin (1µM), fluorocarbonyl cyanide phenylhydrazone (FCCP, 2µM) and rotenone + antimycin A (0.5µM) (Agilent). Analyses were performed on Wave software (Agilent).

References

1. Chatterjee, D. *et al.* Avid binding by B cells to the Plasmodium circumsporozoite protein repeat suppresses responses to protective subdominant epitopes. *Cell Rep.* **35**, 108996 (2021).
2. Ollion, J., Cochennec, J., Loll, F., Escudé, C. & Boudier, T. TANGO: a generic tool for high-throughput 3D image analysis for studying nuclear organization. *Bioinformatics* **29**, 1840–1841 (2013).
3. Sage, P. T. *et al.* Suppression by TFR cells leads to durable and selective inhibition of B cell effector function. *Nat. Immunol.* **17**, 1436–1446 (2016).
4. Ricker, E. *et al.* Serine-threonine kinase ROCK2 regulates germinal center B cell positioning and cholesterol biosynthesis. *J. Clin. Invest.* **130**, 3654–3670 (2020).
5. Xu, H. *et al.* Regulation of bifurcating B cell trajectories by mutual antagonism between transcription factors IRF4 and IRF8. *Nat. Immunol.* **16**, 1274–1281 (2015).
6. Argüello, R. J. *et al.* SCENITH: A Flow Cytometry-Based Method to Functionally Profile Energy Metabolism with Single-Cell Resolution. *Cell Metab.* **32**, 1063-1075.e7 (2020).
