# Supplementary material for: Dynamic mitochondrial transcription and translation in B cells control germinal center entry and lymphomagenesis
Source: Nat Immunol. 2023 Apr 24;24(6):991–1006. doi: 10.1038/s41590-023-01484-3 (PMC10232359; doi:10.1038/s41590-023-01484-3)
Supplement: Supplementary file 2 — Reporting Summary [file 41590_2023_1484_MOESM2_ESM.pdf]

nature portfolio

Corresponding author(s): Alex Clarke NI-A34325B

Last updated by author(s): Mar 5, 2023

Reporting Summary

Nature Portfolio wishes to improve the reproducibility of the work that we publish. This form provides structure for consistency and transparency in reporting. For further information on Nature Portfolio policies, see our Editorial Policies and the Editorial Policy Checklist.

Statistics

For all statistical analyses, confirm that the following items are present in the figure legend, table legend, main text, or Methods section.

n/a	Confirmed	
☐	☒	The exact sample size (*n*) for each experimental group/condition, given as a discrete number and unit of measurement
☐	☒	A statement on whether measurements were taken from distinct samples or whether the same sample was measured repeatedly
☐	☒	The statistical test(s) used AND whether they are one- or two-sided<br>*Only common tests should be described solely by name; describe more complex techniques in the Methods section.*
☐	☒	A description of all covariates tested
☐	☒	A description of any assumptions or corrections, such as tests of normality and adjustment for multiple comparisons
☐	☒	A full description of the statistical parameters including central tendency (e.g. means) or other basic estimates (e.g. regression coefficient) AND variation (e.g. standard deviation) or associated estimates of uncertainty (e.g. confidence intervals)
☐	☒	For null hypothesis testing, the test statistic (e.g. *F*, *t*, *r*) with confidence intervals, effect sizes, degrees of freedom and *P* value noted<br>*Give P values as exact values whenever suitable.*
☒	☐	For Bayesian analysis, information on the choice of priors and Markov chain Monte Carlo settings
☒	☐	For hierarchical and complex designs, identification of the appropriate level for tests and full reporting of outcomes
☒	☐	Estimates of effect sizes (e.g. Cohen's *d*, Pearson's *r*), indicating how they were calculated

Our web collection on statistics for biologists contains articles on many of the points above.

Software and code

Policy information about availability of computer code

Data collection	Zeiss ZEN (blue edition) v3.4 - Zeiss LSM 980 with Airyscan 2 module<br>BD FACS Diva v8.01 - BD LSR II and Fortessa X20 (4-laser) flow cytometer<br>SpectroFlo v3.0 - Cytek Aurora 5-laser flow cytometer<br>INSPIRE v200.1.620.0 - ImageStream<br>SeaHorse Wave Analyzer Software v2.6 - Seahorse. XFe96 Analyzer<br>10x Genomics Cellranger 6.0.1 for single cell analysis
Data analysis	Zeiss ZEN (blue edition) v3.4 - Image analysis<br>Flow Jo v10 - Flow cytometry<br>SeaHorse Wave Desktop Software v2.6 - SeaHorse analysis<br>ImageJ v1.53t - Image analysis<br>Huygens (for deconvolution) v22.04 - Image analysis<br>IDEAS v6.2 and FCS Express v7 - for ImageStream<br>Prism v9.4 for statistical analysis<br>Single cell RNA sequencing data were analyzed using R 4.1.2, with the following packages: Seurat (v4.0.1), DittoSeq (v1.6.0), SCPA (v0.0.0.9), Scuttle (v1.4), EdgeR (v3.36), scRepertoire (1.4), and the Immcantation suite (doi:10.1093/bioinformatics/btu138, doi:10.1093/bioinformatics/btv359) Code used in analyses is available upon reasonable request.

For manuscripts utilizing custom algorithms or software that are central to the research but not yet described in published literature, software must be made available to editors and reviewers. We strongly encourage code deposition in a community repository (e.g. GitHub). See the Nature Portfolio guidelines for submitting code & software for further information.

Data

Policy information about availability of data

All manuscripts must include a data availability statement. This statement should provide the following information, where applicable:

- Accession codes, unique identifiers, or web links for publicly available datasets
- A description of any restrictions on data availability
- For clinical datasets or third party data, please ensure that the statement adheres to our policy

Single cell RNA sequencing (gene expression and VDJ) data have been deposited on GEO under accession number GSE208021.

Human research participants

Policy information about studies involving human research participants and Sex and Gender in Research.

Reporting on sex and gender	N/A
Population characteristics	N/A
Recruitment	N/A
Ethics oversight	N/A

Note that full information on the approval of the study protocol must also be provided in the manuscript.

Field-specific reporting

Please select the one below that is the best fit for your research. If you are not sure, read the appropriate sections before making your selection.

☒ Life sciences ☐ Behavioural & social sciences ☐ Ecological, evolutionary & environmental sciences

For a reference copy of the document with all sections, see nature.com/documents/nr-reporting-summary-flat.pdf

Life sciences study design

All studies must disclose on these points even when the disclosure is negative.

Sample size	No statistical methods were used to pre-determine sample sizes, but our sample sizes are similar to those reported in previous publications. (PMID: 29326381). Effect size for some experiments has been estimated via pilot experiments. The distribution of data was determined using normality testing to determine appropriate statistical methodology, or otherwise assumed to be normally distributed.
Data exclusions	Mice with complete absence of germinal centers as well as lack of Alum spots after immunisation were considered as failed intraperitoneal immunisation and therefore excluded from analysis.
Replication	All experiments included biological and technical replicates. Each experiment reflects at least two independent replicates. All experiments included in the manuscript were reproducible and representative.
Randomization	For in vivo experiments we matched the sex and age of the mice in experimental batches. Care was extra taken to minimise cage effect and litter effect by co-housing control and experimental mice and using littermate controls where it was possible. Randomization measures performed for in vivo experiments were further carried over into in vitro and ex vivo experiments, which were set up with cells isolated from wild type or knock out mice. During sample acquisitions experimental and control samples were run consecutively in alternating fashion. Other modes of randomization was not performed as they were not relevant for our study.
Blinding	For some experiments, researchers were blinded, for example where mice were genotyped after the experiment was completed. For monitoring disease development in lymphoma-prone mice, animal facility technicians played a role as blinded observers. Data collection and analysis were not performed blind to the conditions of the experiments in most of the experiments because these experiments were performed by the same researchers and the mouse genotype information needed to know to ensure employment of both wild type and knock out mice in the study.

Reporting for specific materials, systems and methods

We require information from authors about some types of materials, experimental systems and methods used in many studies. Here, indicate whether each material, system or method listed is relevant to your study. If you are not sure if a list item applies to your research, read the appropriate section before selecting a response.

Materials & experimental systems

n/a	Involved in the study
☐	☒ Antibodies
☐	☒ Eukaryotic cell lines
☒	☐ Palaeontology and archaeology
☐	☒ Animals and other organisms
☒	☐ Clinical data
☒	☐ Dual use research of concern

Methods

n/a	Involved in the study
☒	☐ ChIP-seq
☐	☒ Flow cytometry
☒	☐ MRI-based neuroimaging

Antibodies

Antibodies used

Flow cytometry antibodies

Antibody Clone Manufacturer Cat no:
Anti-CD45.2 104 BioLegend 109841
Anti-CD45.1 A20 BioLegend 110718
Anti-CXCR4 REA107 Miltenyi Biotec 130-123-274
Anti-CD19 6D5 Biolegend 115545
Anti-B220 RA3-6B2 Biolegend 103247
Anti-CD38 90 Biolegend 102717
Anti-CD138 281-2 Biolegend 142507
Anti-CD138 281-2 Biolegend 142525
Anti-CD86 GL-1 Biolegend 105031
Anti-CD86 GL-1 Biolegend 105043
Anti-CXCR4 2B11 Thermo Fisher 48-9991-82
Anti-IgD 11-26c.2a Biolegend 405723
Anti-IgD 11-26c.2a Biolegend 405721
Anti-IgM RMM-1 Biolegend 406513
Anti-CD21/35 7E9 Biolegend 123409
Anti-BP-1 6C3 Miltenyi Biotec 130-102-181
Anti-CD23 B3B4 Biolegend 101621
Anti-CD43 S11 Biolegend 143205
Anti-CD24 M1/69 Biolegend 101819
Anti-GL-7 GL7 Biolegend 144605
Anti-GL-7 GL7 Biolegend 144611
Anti-GL-7 GL7 Biolegend 144613
Anti-Gr1 RB6-8C5 Biolegend 108423
Anti-CD3 17A2 Biolegend 100221
Anti-CD4 RM4-5 Biolegend 100553
Anti-CD19 6D5 Biolegend 115537
Anti-CXCR5 L138D7 Biolegend 145503
Anti-IgG1 RMG1-1 Biolegend 406629
Anti-ICOS C398.4A Biolegend 313549
Anti-GITR DTA-1 Biolegend 126311
Anti-cyclin B1 V152 Biolegend 647905
Anti-cleaved caspase 3 (Asp175) 9661 Cell signaling 9661T
Anti-phospho histone 3 (Ser10) D2C8 Cell signaling 3465S
Anti-SDHB 21A11AE7 Abcam ab197722
Anti-COX I 1D6E1A8 Abcam ab154477
Anti-TOMM20 EPR15581-54 Abcam ab186735
Anti-TFAM EPR23548-120 Abcam ab252432
Anti-LAMP1 EPR21026 Abcam ab237307
Anti-MCU D2Z3B Cell Signaling 14997S
Anti-CD16/32 (BD mouse Fc Block) 2.4G2 BD 553142
NP-PE Biosearch N-5070-1
NP-APC in-house in-house
Goat Anti-Rabbit IgG (H&L) SuperClonal antibody ThermoFisher A27034
Goat Anti-Rabbit IgG (H&L) SuperClonal antibody ThermoFisher A55055
Donkey anti-rabbit IgG (minimal x-reactivity) Antibody BioLegend 406421

Spectral flow cytometry antibody panel

Antibody Clone Manufacturer Cat no:

Anti-CD19 6D5 BioLegend 115545
Anti-CD21/35 7E9 Biolegend 123421
Anti-CD93 AA4.1 BD Bioscience 741989
Anti-IgD 11-26c.2a Biolegend 405721
Anti-CD4 RM4-5 Biolegend 100547

Anti-IgM II/41 BD Bioscience 743329
Anti-CD23 B3B4 BD Bioscience 563988
Anti-B220 RA3-6B2 Biolegend 103247
Anti-CD19 6D5 Biolegend 115543
Anti-CD24 M1/69 BD Bioscience 563450
Anti-BP-1 (CD249) 6C3 BD Bioscience 741743
Anti-CD43 S7 BD Bioscience 747726
Anti-CXCR4 2B11 BD Bioscience 741783
Anti-CD86 GL-1 Biolegend 105043
Anti-CD38 90/CD38 (Ab90) BD Bioscience 741955
Anti-CD138 281-2 Biolegend 142519
Anti-CD4 RM4-5 Biolegend 100547
Anti-GL-7 GL7 Biolegend 144609
Anti-TFAM EPR23548-120 Abcam ab252432
Complex I Monoclonal Antibody 18G12BC2 Invitrogen 43-8800
Anti-Cytochrome C 7H8.2C12 Novus Bio NB100-56503AF700
Anti-COX I (Complex IV) 1D6E1A8 Abcam ab198600
Anti-COX IV 3C7D2 Proteintech CL488-60251
Anti-SDHA 2E3GC12FB2AE2 Abcam ab170172
Anti-ATP5A1 1B10H3 Proteintech CL555-66037
Anti-HSP60 2F10E7 Proteintech CL594-66041
Anti-Grp75/mtHSP70 OTI9F8 Novus Bio NBP1-47801
Zombie fixable viability dye Biolegend 423105
Anti-CD16/32 (FcBlock) 2.4G2 BD 553142
Goat anti-Rabbit IgG (H+L) Highly Cross-Adsorbed Secondary Antibody ThermoFisher A48254

IHC & ICC

Antibody Clone Manufacturer Cat no:
Anti-CD38 90 Biolegend 102716
Anti-CD21/35 7E9 Biolegend 123423
Anti-CD21/35 7E9 Biolegend 123407
Anti-CD138 281-2 Biolegend 142525
Anti-IgD 11-26c.2a Biolegend 405707
Anti-GL-7 GL-7 Biolegend 144605
Anti-GL-7 GL-7 Biolegend 144611
Anti-GL-7 GL-7 Biolegend 144613
CD3e Monoclonal Antibody 500A2 eBioscience 14-0033-82
Anti-SDHB 21A11AE7 Abcam ab197722
Anti-COX I 1D6E1A8 Abcam ab198600
Anti-TOMM20 EPR15581-54 Abcam ab186735
Anti-Lonp1 Polyclonal Atlas HPA002192
Anti-TFAM EPR23548-120 Abcam ab252432
Goat Anti-Hamster IgG (H+L)-0.5 mL Thermofisher A-21451
Goat Anti-Rabbit IgG (H&L) SuperClonal antibody ThermoFisher A27034
Goat Anti-Rabbit IgG (H&L) SuperClonal antibody ThermoFisher A55055

Validation

All antibodies were ordered from commercial vendors, which validated antibodies (Biolegend, BD Bioscience, eBioscience, Miltenyi, Abcam, Cell signalling). When applicable, we performed further validation using knock-out mice or relevant isotype controls. Please refer to the manufacturer's website using the catalog numbers listed above should the validation information of a particular antibody need to be viewed. Further information can be obtained from the vendor's websites:

https://www.miltenyibiotec.com/GB-en/products/macs-antibodies/antibody-validation.html?countryRedirected=1
https://www.bdbiosciences.com/en-gb/products/reagents/flow-cytometry-reagents/research-reagents/quality-and-reproducibility
https://www.biolegend.com/en-us/quality/quality-control
https://www.abcam.com/primary-antibodies/a-guide-to-antibody-validation
https://www.ptglab.com/
https://www.cellsignal.com/learn-and-support/videos-and-webinars/cst-antibody-validation-documentary?gclid=CjwKCAiAmJGgBhAZEiwA1JZolqXP7c01OXJ8shE-lMEmfmQIzVesF22qol99dmdWL4DEEgZACFKj5hoCyLsQAvD_BwE&gclsrc=aw.ds

Below we provided the full antibody list with the reference or validation details from vendor or our lab.

Antibody Clone Manufacturer Validation/Reference
Anti-CD45.2 104 75 BioLegend PMID: 32103173
Anti-CD45.2 104 100 BioLegend PMID: 33765443
Anti-CD45.1 A20 100 BioLegend PMID: 33765443
Anti-CD45.1 A20 100 BioLegend PMID: 33046889
Anti-CXCR4 REA107 Miltenyi PMID: 25344471
Anti-CD19 6D5 Biolegend PMID: 30257198
Anti-B220 RA3-6B2 Biolegend PMID: 31412246
Anti-CD38 90 Biolegend PMID: 31810882
Anti-CD138 281-2 Biolegend PMID: 30538335
Anti-CD138 281-2 Biolegend PMID: 31618654
Anti-CD86 GL-1 Biolegend PMID: 34157302

Anti-CD86 GL-1 Biolegend PMID: 34343496
Anti-CXCR4 2B11 Thermofisher PMID: 33432228
Anti-IgD 11-26c.2a Biolegend PMID: 33010224
Anti-IgD 11-26c.2a Biolegend PMID: 29752062
Anti-IgM RMM-1 Biolegend PMID: 33278339
Anti-CD21/35 7E9 Biolegend PMID: 28841417
Anti-BP-1 6C3 Miltenyi Biotec PMID: 2809203
Anti-CD23 B3B4 Biolegend PMID: 34214192
Anti-CD43 S11 Biolegend PMID: 30270123
Anti-CD24 M1/69 Biolegend PMID: 20720183
Anti-GL-7 GL7 Biolegend PMID: 29752062
Anti-GL-7 GL7 Biolegend PMID: 31563464
Anti-GL-7 GL7 Biolegend PMID: 29221730
Anti-Gr1 RB6-8C5 Biolegend PMID: 30538335
Anti-CD3 17A2 Biolegend PMID: 27641500
Anti-CD4 RM4-5 Biolegend PMID: 30737144
Anti-CD19 6D5 Biolegend PMID: 30770250
Anti-CXCR5 L138D7 Biolegend PMID: 34860581
Anti-IgG1 RMG1-1 Biolegend Application FC - Quality tested (Biolegend)
Anti-ICOS C398.4A Biolegend PMID: 35202565
Anti-GITR DTA-1 Biolegend PMID: 30611611
Anti-cyclin B1 V152 Biolegend PMID: 36189922
Anti-cleaved caspase 3 (Asp175) 9661 Cell signaling PubMed ID: 36658493
Anti-phospho histone 3 (Ser10) D2C8 Cell signaling PMID: 31131319

Anti-SDHB 21A11AE7 Abcam Knockout validated by Abcam
Anti-COX I 1D6E1A8 Abcam PMID: 33238133 / further validated in Tfam-KO mice and chloramphenicol treated cells
Anti-TOMM20 EPR15581-54 Abcam PMID: 33882315
Anti-TFAM EPR23548-120 Abcam "Suitable for: IP, Flow Cyt (Intra), WB, ICC/IF (abcam)" / further validated in Tfam KO mice using ICC, IHC-Fr and flow cytometry / showed strong colocalisation with other mitochondrial markers in WT cells
Anti-LAMP1 EPR21026 Abcam "Suitable for: ICC/IF" by Abcam (used in imaging flow cytometry experiments)
Anti-MCU D2Z3B Cell Signaling Quality tested for IF by the vendor, further validated in Tfam-KO B cells which shows upregulated MCU and T cells (WT) from same mice being the negative control

Anti-CD16/32 (BD mouse Fc Block) 2.4G2 BD PMID: 11709085
NP-PE Biosearch Validated on unimmunised tissue/mice
NP-APC in-house Validated on unimmunised tissue/mice
Goat Anti-Rabbit IgG (H&L) SuperClonal antibody ThermoFisher N/A
Goat Anti-Rabbit IgG (H&L) SuperClonal antibody ThermoFisher N/A

Spectral flow cytometry antibody panel

Antibody Clone Manufacturer Validation/Reference

Anti-CD19 6D5 BioLegend PMID: 30257198
Anti-CD21/35 7E9 Biolegend PMID: 33296685
Anti-CD93 AA4.1 BD Bioscience PMID: 11739500
Anti-IgD 11-26c.2a Biolegend PMID: 29752062
Anti-CD4 RM4-5 Biolegend PMID: 29150240
Anti-IgM II/41 BD Bioscience PMCID: PMC22519
Anti-CD23 B3B4 BD Bioscience PMID: 10508270
Anti-B220 RA3-6B2 Biolegend PMID: 29942093
Anti-CD19 6D5 Biolegend PMID: 28636954
Anti-CD24 M1/69 BD Bioscience PMID: 9075925
Anti-BP-1 (CD249) 6C3 BD Bioscience PMID: 12374812
Anti-CD43 S7 BD Bioscience PMID: 1827140
Anti-CXCR4 2B11 BD Bioscience PMID: 9570576
Anti-CD86 GL-1 Biolegend PMID: 34157302
Anti-CD38 90/CD38 (Ab90) BD Bioscience PMID: 9694721
Anti-CD138 281-2 Biolegend PMID: 32213346
Anti-CD4 RM4-5 Biolegend PMID: 29150240
Anti-GL-7 GL7 Biolegend PMID: 34914544
Anti-TFAM EPR23548-120 Abcam "Suitable for: IP, Flow Cyt (Intra), WB, ICC/IF (abcam)" / further validated in Tfam KO mice using ICC, IHC-Fr and flow cytometry / showed strong colocalisation with other mitochondrial markers in WT cells
Complex I Monoclonal Antibody 18G12BC2 Invitrogen Validated for flow cytometry by the vendor
Anti-Cytochrome C 7H8.2C12 Novus Bio "Applications WB, Flow, ICC/IF, IHC, IHC-P " from vendor
Anti-COX I (Complex IV) 1D6E1A8 Abcam Validated in Tfam-KO mice and chloramphenicol treated B cells
Anti-COX IV 3C7D2 Proteintech PMID: 33607155
Anti-SDHA 2E3GC12FB2AE2 Abcam "Suitable for: ICC/IF" from abcam / validated using Mito-flow approach in-house
Anti-ATP5A1 1B10H3 Proteintech Validated by vendor
Anti-HSP60 2F10E7 Proteintech Validated by vendor
Anti-Grp75/mtHSP70 OTI9F8 Novus Bio "Applications: WB, Flow, ICC/IF, IHC, IHC-P" from vendor's website
Zombie fixable viability dye Biolegend
Anti-CD16/32 (FcBlock) 2.4G2 BD PMID: 11709085
Goat anti-Rabbit IgG (H+L) Highly Cross-Adsorbed Secondary Antibody ThermoFisher

IHC & ICC

Antibody Clone Manufacturer Validation/Reference
Anti-CD38 90 Biolegend Validated by vendor
Anti-CD21/35 7E9 Biolegend Validated by vendor
Anti-CD21/35 7E9 Biolegend Validated in house via confocal imaging of FDC
Anti-CD138 281-2 Biolegend Validated in house via confocal imaging using Blimp-1 mVenus reporter mouse
Anti-IgD 11-26c.2a Biolegend Validated by vendor
Anti-GL-7 GL-7 Biolegend Validated by vendor
Anti-GL-7 GL-7 Biolegend Validated by vendor
Anti-GL-7 GL-7 Biolegend Validated in house via confocal imaging using Aicda-tdTom reporter mouse
CD3e Monoclonal Antibody 500A2 eBioscience PMID: 31968240
Anti-SDHB 21A11AE7 Abcam Knockout validated by Abcam
Anti-COX I 1D6E1A8 Abcam Validated in Tfam-KO mice and chloramphenicol treated B cells
Anti-TOMM20 EPR15581-54 Abcam PMID: 33882315 / further validated by the vendor
Anti-Lonp1 Polyclonal Atlas Validated in house via TOM20 costaining
Anti-TFAM EPR23548-120 Abcam "Suitable for: IP, Flow Cyt (Intra), WB, ICC/IF (abcam)" / further validated in Tfam KO mice using ICC, IHC-Fr and flow cytometry / showed strong colocalisation with other mitochondrial markers in WT cells

Goat Anti-Hamster IgG (H+L)-0.5 mL Thermofisher
Goat Anti-Rabbit IgG (H&L) SuperClonal antibody ThermoFisher
Goat Anti-Rabbit IgG (H&L) SuperClonal antibody ThermoFisher

Eukaryotic cell lines

Policy information about cell lines and Sex and Gender in Research

Cell line source(s)

Daudi Cell line (kindly gifted by Lynn Dustin, commercially available at https://www.atcc.org/products/ccl-213), 40LB cell line (A fibroblast cell line, BALB/3T3 A31, stably transfected with expression vectors encoding mouse CD40-ligand (CD40L) and BAFF, kindly gifted by Daisuke Kitamura, commercially available at https://cellbank.brc.riken.jp/cell_bank/CellInfo/?cellNo=RCB5304&lang=En)

Authentication

Morphology was assessed by microscopy and presence of B-cell lineage markers (Daudi) and CD40L expression (40LB) by flow cytometry

Mycoplasma contamination

The cell lines were not tested for mycoplasma in our lab.

Commonly misidentified lines
(See ICLAC register)

No commonly misidentified cell lines used in this study

Animals and other research organisms

Policy information about studies involving animals; ARRIVE guidelines recommended for reporting animal research, and Sex and Gender in Research

Laboratory animals

B6.Cg-Tfamtm1.1Ncdl/J (JAX:026123), B6.C(Cg)-Cd79atm1(cre)Reth/EhobJ (JAX: 020505), B6.129P2-Aicdatm1(cre)Mnz/J (JAX:007770), B6;129S6-Gt(ROSA)26Sortm9(CAG-tdTomato)Hze/J (Ai9, JAX:007905), B6.Cg-Tg(TcraTcrb)425Cbn/J (JAX: 004194) and B6.Cg-Tg(IghMyc)22Bri/J [Eμ-Myc] (JAX: 002728) were purchased from Jackson Laboratories. Tg(Prdm1-Venus)1Sait [Blimp1-mVenus] (MGI:3805969) was a kind gift from Mitinori Saitou (Kyoto University). Gt(ROSA)26Sortm1(CAG-mCherry/GFP)Ganl (MitoQC) was a kind gift from Ian Ganley (University of Dundee). B6.SJL.CD45.1 mice were provided by central breeding facility of the University of Oxford. Male and female mice between the ages of 6-15 weeks were used. Mice were bred and maintained under specific pathogen-free conditions at the Kennedy Institute of Rheumatology, University of Oxford. All procedures and experiments were performed in accordance with the UK Scientific Procedures Act (1986) under a project license authorized by the UK Home Office (PPL number: PP1971784). The mice underwent regular checks to ensure they did not have any pathogenic microorganisms. They were housed in cages that had individual ventilation and were provided with things to stimulate their environment. The temperature was kept between 20-24°C, with a humidity level of 45-65%. They were exposed to a 12-hour cycle of light and darkness (7 am to 7 pm), with a thirty-minute period of dawn and dusk.

Wild animals

No wild animals employed in this study.

Reporting on sex

Our findings are applicable to both male and female mice and we did not observe gender-effect throughout our study. Therefore our study design did not restrict the usage of particular sex. Please see the randomisation section above for our randomisation strategy.

Field-collected samples

The study did not involve any field-collected samples

Ethics oversight

Mice were bred and maintained under specific pathogen-free conditions at the Kennedy Institute of Rheumatology, University of Oxford. All procedures and experiments were performed in accordance with the UK Scientific Procedures Act (1986) under a project license authorized by the UK Home Office (PPL number: PP1971784)

Note that full information on the approval of the study protocol must also be provided in the manuscript.

Flow Cytometry

Plots

Confirm that:

☒ The axis labels state the marker and fluorochrome used (e.g. CD4-FITC).

☒ The axis scales are clearly visible. Include numbers along axes only for bottom left plot of group (a 'group' is an analysis of identical markers).

☒ All plots are contour plots with outliers or pseudocolor plots.

☒ A numerical value for number of cells or percentage (with statistics) is provided.

Methodology

Sample preparation

Flow cytometry and imaging flow cytometry

Briefly, harvested spleens were injected with ice cold PBS and mashed through a 70µm strainer (Falcon) or crushed between the frosted ends of microscope slides. For Peyer's patch dissociation, a 40µm strainer (VWR) was used. RBCs were depleted by incubating splenocytes with ACK Lysis Buffer (Gibco) for 3-4 mins at 20°C. Single cell suspensions were incubated with Fixable Viability Dye eFluor™ 780 (eBioscience) in PBS, followed by FcBlock (5 mins) and surface antibodies (30 mins on ice) in FACS buffer (PBS supplemented with 0.5% BSA and 2mM EDTA). For intracellular staining, cells were fixed at 20°C with 4% freshly prepared PFA (Cell Signaling) for 15 mins and permeabilized with methanol (90% ice cold for 10 mins on ice with frequent vortexing) unless specified otherwise. Phalloidin-based F-Actin (Thermofisher, cat no: A30107) staining was performed using BD Perm/Wash reagent (BD, Cat. No. 554723) following 4% PFA fixation. For in vivo cell cycle analysis, 5-ethynyl-2'-deoxyuridine (EdU) (1 mg, Thermofisher, cat no: A10044) was injected intraperitoneally and mice were sacrificed after 2.5 h. Cells were stained for surface markers, fixed and permeabilized then labelled using Click chemistry according to manufacturer's instructions (Click iT Plus EdU Flow cytometry kit, Thermo Fisher, cat: C10634). FxCycle Violet (Thermo Fisher, cat: F10347) reagent was used for cell cycle characterization. For mitochondrial superoxide deep red (mtSOX, Dojindo) uptake, following viability dye and surface staining, cells were resuspended in warm complete RPMI supplemented with mtSOX (10µM) and incubated for 30 mins at 37°C. Cell were washed twice before flow cytometry acquisition. Flow cytometry was performed on BD Fortessa X-20 or LSR II instruments (both BD), or using a Cytek Aurora (5 laser) spectral flow cytometer. For imaging cytometry, single cell suspensions were prepared from spleens of MitoQC mice, incubated with live-dead dye, stained for surface markers, and then fixed with 4% PFA. Washed cells were then resuspended in 50µl FACS Buffer and run on an Amnis ImageStreamX Mark II Imaging Flow Cytometer and analyzed with IDEAS (EMD Millipore) and FCS Express software (v7.12). Flow cytometry data was analyzed using FlowJo (BD).

Detection of ETC/UPRmt proteins with spectral flow cytometry

A complete list of antibodies is shown in the antibody table. All antibodies targeting intracellular mitochondrial proteins were directly conjugated and of mouse origin, with the exception of rabbit anti-Tfam monoclonal antibody (Abcam). Goat anti-mouse AF405 Plus secondary antibody (ThermoFisher) was used for detection. All antibodies used in the panel had been either validated for flow cytometry by the vendor or in the literature. Mouse anti-mitochondrial complex 1 antibody was conjugated in house using a PE-Cy7 Lightning-link kit (Novus Bio, cat: 762-0005). Cells were labelled with Zombie NIR viability dye (Biolegend) and Fcblock in PBS for 30 min on ice in 96-well V-bottom plates. Following washing, surface staining was performed in Brilliant staining buffer (BD, cat: 563794) for 30 min. Cells were then fixed in 4% freshly-made PFA at 20°C for 15 mins and permeabilized in freezer-cold methanol for 10 mins with occasional vortexing. Anti-Tfam primary staining was performed in 50µl FACS Buffer supplemented with 2% goat serum for 45 mins at 20°C. Following two washes, goat anti-rabbit secondary antibody and the remaining directly conjugated antibodies for ETC/UPRmt proteins were added for 30 mins at 20°C. The cells were subsequently washed in FACS buffer and acquired on a Cytek Aurora (5 laser) spectral flow cytometer. Exploratory pilot experiments were performed to determine the most suitable single-stained references (beads or cells) for individual marker-fluorochrome combinations. If cells were used as reference controls, they were obtained from matching organs (spleen or bone marrow). Reference controls were processed similarly to fully stained samples, with parallel fixation, permeabilization, and washing steps. Acquired samples were unmixed using SpectroFlow and analyzed with FlowJo. The 'Autofluorescence (AF) as a fluorescent tag' option was enabled during unmixing to minimize AF interference. In some rare cases minor adjustments were applied to unmixing on the SpectroFlow software. gMFI values for ETC/UPRmt proteins were calculated using FlowJo.

Instrument

BD Fortessa X20, BD LSR II, BD Aria, Cytek Aurora (5 laser)

Software

FlowJo v10, Spectroflo v3

Cell population abundance

Cell sorting

Naïve B cells were isolated using the Pan B cell Isolation II Kit, anti-CD43, and/or anti-CD23 Microbeads (all Miltenyi) according to the manufacturer's instructions. Purity validated by flow cytometry was >90%. Isolation of DZ-LZ-GZ subsets of GC B cells, and TFH cells (CD19- CD4+ CXCR5+ ICOS+ GITR-) was performed via fluorescence-activated cell sorting (FACS).

For some experiments, untouched GC B cells were isolated using a magnetic bead-based protocol as described.55. Briefly, spleens were harvested from SRBC-immunized mice, and single cell suspensions were prepared in ice cold MACS isolation buffer (PBS with 0.5% BSA + 2mM EDTA) followed by ACK RBC lysis (Gibco) for 4 mins at 20°C with occasional mixing every

30s. After washing, cells were labelled with anti-CD43 microbeads (Miltenyi) and biotinylated antibodies against CD38 and CD11c (both eBioscience, clones 90 and N418 respectively), followed by incubation with anti-biotin microbeads, and subsequently run through a MACS LS column (Miltenyi). Purity was confirmed by flow cytometry and immunocytochemistry (ICC) and exceeded 95%.
When sorting for single cell RNA sequencing, spleens were crushed using the rough ends of microscope slides to maximize cell yield. Subsequently, non-B cells were depleted using the Pan B cell Kit II (Miltenyi). Enriched cells were then incubated with viability dye, anti-CD16/32 (FcBlock) and surface flow antibodies, including markers for an exclusion (dump) channel (anti-CD3, anti-Gr1 and anti-CD11c), and live Dump- tdTomato+ cells were sorted by BD Aria FACS. When flow sorting was performed, the purity was carefully monitored and each case it was above 90%.

Gating strategy

ymphocytes were initially gated based on FSC/SSC properties followed by exclusion of doublets. Then, viable cells were gated by excluding Live/Dead+ cells. Further downstream gating strategy was indicated in main and supplementary figures/legends

☒ Tick this box to confirm that a figure exemplifying the gating strategy is provided in the Supplementary Information.
